# Prevalence and Determinants of Depression Among Multi-Drug Resistant (MDR) TB Cases

**DOI:** 10.1192/bjo.2026.11078

**Published:** 2026-06-30

**Authors:** Muhammad Ayub, Aliya Khan, Darshana Kumari

**Affiliations:** 1 Pak International Hospital, Karachi, Pakistan; 2 Northamptonshire Healthcare NHS Foundation Trust, Northamptonshire, United Kingdom; 3 Black Country Healthcare NHS Foundation Trust, West Midlands, United Kingdom

## Abstract

**Aims:**

Multidrug-resistant tuberculosis (MDR-TB) is associated with prolonged treatment, significant psychosocial stressors, and poor treatment outcomes. Depression is a common but under-recognised comorbidity in this population and may negatively affect adherence and recovery. This study aimed to determine the prevalence of depression among patients with MDR-TB and to identify socio-demographic and clinical factors associated with depression. We hypothesised that depression would be highly prevalent among MDR-TB patients and associated with financial stress, family problems, longer treatment duration, and increasingage.

**Methods:**

A descriptive cross-sectional study was conducted at a tertiary care hospital in Karachi, Pakistan. A total of 107 adult patients diagnosed with MDR-TB were recruited using non-probability consecutive sampling. Patients who had been receiving anti-tuberculosis treatment for more than two months were included. Pregnant patients, those receiving psychiatric treatment for conditions other than depression, and those unwilling to participate were excluded. Depression was assessed using the Patient Health Questionnaire-9 (PHQ-9). A score greater than 4 was considered indicative of depressive symptoms. Data were collected on age, gender, education, employment status, duration of treatment, financial difficulties, and family problems related to the TB diagnosis. Statistical analysis was performed using SPSS version 20. Associations between depression and potential determinants were analysed using the Chi-square test, with a p-value of ≤0.05 considered statistically significant.

**Results:**

The mean age of participants was 32.1 ± 6.2 years, with a predominance of male patients (69.2%). Depression was identified in 52 patients (48.6%). Depression was significantly associated with increasing age (p=0.001), financial difficulties (p=0.01), family problems (p=0.01), and longer duration of MDR-TB treatment (p=0.01). Patients undergoing treatment for more than eight months demonstrated the highest prevalence of depressive symptoms. No significant associations were observed with gender, education level,or employment status.

**Conclusion:**

Nearly half of patients with MDR-TB in this study experienced depressive symptoms, highlighting a substantial mental health burden in this population. Financial stress,family difficulties, prolonged treatment duration, and increasing age were key determinants of depression. These findings underscore the importance of routine mental health screening and integrated psychosocial support within MDR-TB treatment programmes to improve adherence, treatment outcomes, and overall quality of life.

